# Small-scale implementation with pragmatic process evaluation: a model developed in primary health care

**DOI:** 10.1186/s12875-018-0778-6

**Published:** 2018-06-21

**Authors:** Kirsti Malterud, Aase Aamland, Kristina Riis Iden

**Affiliations:** 1 Research Unit for General Practice, Uni Research Health, Uni Research, Kalfarveien 31, N-5018 Bergen, Norway; 2 The Research Unit for General Practice and Section of General Practice, Department of Public Health, University of Copenhagen, Copenhagen, Denmark; 3 Department of Global Public Health and Primary Care, University of Bergen, Bergen, Norway

**Keywords:** Implementation model, Process evaluation, Evidence, Primary health care, Depression, Nursing home

## Abstract

**Background:**

Research often fails to impose substantial shifts in clinical practice. Evidence-based health care requires implementation of documented interventions, with implementation research as a science-informed strategy to identify core experiences from the process and share preconditions for achievement. Evidence developed in hospital contexts is often neither relevant nor feasible for primary care. Different evidence types may constitute a point of departure, stretching and testing the transferability of the intervention by piloting it in primary care. Comprehensive descriptions of aims, context and procedures can be a more useful outcome than traditional effect studies.

**Main text:**

We present a model for small-scale implementation of relevant research evidence, monitored by pragmatic evaluation. The model, which is applicable in primary care, is supported by Weiner’s theory about organizational readiness for change and consists of four steps: 1) recognize the problem – identify a workable intervention, 2) assess the context – prepare for inception, 3) pilot the intervention on site, and 4) upscale and accomplish the intervention. The process is evaluated by exploring selected relevant aspects of experiences and outcomes from the first to the last step. Process evaluation is a logical precondition for outcome evaluation – attempting to assess either the efficacy or the effectiveness of a “black box” intervention makes no sense. We argue why evidence beyond effect studies and evaluation beyond randomized controlled trials may be adequate for science-informed evaluation of a small-scale implementation project such as is often conducted by primary health care practitioners. The model is illustrated by an ongoing project, in which a strategy for upgrading the management of depression in nursing homes in Norway is currently being implemented.

**Conclusions:**

A flexible and manageable approach is suggested, in which the inevitable unpredictability of clinical practice is incorporated. Finding the appropriate middle ground between rigour and flexibility, some compromises must be made. Our model recognizes the skills of practical knowing as something other than traditional medical research, while maintaining academic values such as systematic and transparent reflection, using adequate tools. Considering the purpose and context of our model, we argue that these priorities, emphasizing relevance and feasibility, are strengths, not limitations.

## Background

Medical research is conducted to make an impact on health and disease, and health care services are increasingly adopting *evidence-based health care (EBHC)* [1]*.* Still, a substantial proportion of research evidence finds its way into scientific journals and languishes there without ever leading to substantial shifts in clinical practice [2]. Pathirana et al. describe how different drivers cause over-diagnosis, such as the promotion of increasingly sensitive tests, leading to the detection of minor “abnormalities”, which may be of uncertain clinical significance [3]. While there is evidence for substantial over-diagnosis of thyroid cancer (for women in the Nordic countries it is estimated at 50%; [4]), diagnostic procedures remain unchanged.

*Implementation* is a process in which we transform what we know to what we do, monitored by *implementation research* to evaluate conditions influencing these processes [5, 6]. Implementation research offers tools with which to explore and reflect upon what is likely to work in this situation for these people in this organization with these constraints [1]. Thus, the description and analysis of experiences from an intervention process in a specific context may offer inspiration and wisdom for transformation to analogous real-life environments.

The *primary care context* presents specific challenges to EBHC. A large proportion of knowledge and skills from this medical domain is not yet or will never be substantiated by research evidence, simply because it is fluid, flexible and individualized [2, 7, 8]. Moreover, evidence developed in a top-down hospital context may be neither relevant nor feasible for primary care, owing to the different prevalence, distribution and nature of the health problems that occur at these different levels. Critical conditions related to structural framework, legal regulations, resources, professional procedures and social interaction also often differ between primary care and hospital care [9].

*Clinical guidelines* for diagnosis, treatment and prevention are the most common format for the implementation of EBHC. Although general practitioners (GPs) seem to approve guidelines in general, several barriers obstruct their application in the real world [10–12]. Flottorp et al., for example, tried to implement guidelines for the management of two common disorders in general practice, but even tailored interventions targeting identified barriers failed to change practice [13].

It is neither advisable nor realistic to regulate any clinical procedure with guidelines. Still, numerous demands for quality improvement remain unmet and significant emerging, potentially relevant evidence is never translated into practice [5]. Greenhalgh draws attention to the huge gap between evidence and its implementation, pointing out that these issues are complex and not easily explained [1].

How, then, can this state of affairs be rectified [2]? In this article, we aim to promote reflection and motivation for change by presenting a model for the small-scale *implementation* of relevant research evidence applicable in primary care, monitored by *implementation research* conducted as pragmatic evaluation.

## Main text

### Theoretical framework and basic concepts

Plenty of relevant theories are available that attempt to explain how and why people and organizations may take up innovations and change their behaviour [1]. We have chosen Weiner’s theory about *Organizational Readiness for Change (ORC)* [14] to support our model, because it is simple while still including a few crucial concepts emphasizing the organizational framework to which participating individuals belong. Translation of knowledge into practice entails ORC, determined by two dimensions: 1) whether organizational members value the intended change and think that it is worthwhile and important (*change commitment*), and 2) how organizational members appraise the demands of the particular task, resource availability, requirements for the task and situational factors (*change efficacy*). The appraisal of selected and relevant ORC aspects is indispensable to planning and implementation. These aspects are also important targets for the evaluation of an implementation process.

We argue that implementation of EBHC should always be accompanied by implementation research, to identify core experiences from the process and share preconditions for achievement with colleagues. For analytical purposes, the two different but mutually interacting levels of *doing* and *the study of what is being done* must be recognized. Greenhalgh stresses that implementing EBHC involves complex practices that require skills and situational judgment and are not just a matter of following procedural steps [1]. Furthermore, she argues that “implementation science” is neither science nor art, but a *science-informed practice*. Greenhalgh points out that traditional health care research is oriented towards producing statistical generalizations based on a sample from one population to predict what will happen in a comparable population, leading to one single interpretation of the findings. In contrast, she says, implementation science is at least partly about using unique case examples as a window opening onto wider truths, through the enrichment of understanding, with multiple possible interpretations of a case. Both doing and the study of what is being done therefore require conscientious modification to be feasible and relevant in the actual context [6].

*Implementation* of research evidence into practice is a complex process, calling for methodological skills as well as practical wisdom. Using the EBHC concept, we draw on a base of existing research knowledge to support the intervention we intend to implement. *Evidence* informing the intervention is acquired from qualitative, quantitative or mixed-method studies, depending on the nature of the problem to be solved, the type of intervention to be implemented, the implementation context and available time and resources. Evidence supports the aim, nature and content of the intervention, often also its *efficacy* (outcome under ideal conditions). Complex interventions need to be tailored to local circumstances, rather than being firmly standardized [1, 15]. Feasibility is supported by simplicity and pragmatic priorities, as well as close interaction with the people and the context involved [16]. Bottom-up strategies and implementation anchored in contextual skills and experience are crucial to increase the likely success of the implementation.

*Implementation research* offers tools and concepts for the evaluation of preconditions for the successful implementation of strategies, programmes, interventions or individual practices. Monitoring, assessing and sharing the experiences of facilitators and barriers to the implementation of a certain intervention in a particular context are helpful for colleagues who want to do something similar [16]. Implementation research is a composite realm with a variety of roots [1], for example quality improvement [17], evidence-based medicine (EBM) [18], organizational psychology [19] and evaluation research [20].

### Pragmatic process evaluation – focus and priorities

Pragmatic evaluation is a specific and flexible strategy among several available options, and the concept is not synonymous with implementation research. It is impossible to evaluate every tiny piece of the entire process. A starting point for a relevant and feasible evaluation strategy is to realize that knowledge is always partial, intermediate and dependent on the situated view of the researcher [21]. Choices and priorities must therefore be made about what knowledge will be most helpful for others, given the available resources. *Relevance* to the purpose and context is the overarching yardstick determining which features to select for review in the evaluation plan [20].

At an early stage, you will have to decide upon a primary scope and recipient of your implementation research. Do you want to share your experiences with colleagues who plan to take up similar interventions, are you talking to public health authorities to convince them of the need for and shape of a national plan, or are you speaking to the assessment committee of your Ph.D., who are obliged to maintain academic methodological standards? The format and focus of the report will be different depending on whom it addresses, but all recipients deserve proper, high-quality contributions. An implementation research project can be published as an article in a scientific journal if you follow accepted principles for qualitative or quantitative research and present original, relevant and credible knowledge. Mixed-methods designs may also be useful [22]. The report of a small-scale primary care implementation project may sometimes be more useful for potential recipients if it is not forced into the restricted format of a peer-reviewed article or subjected to traditional expectations of generalizability.

*Process evaluation* (often called *formative evaluation*) aims to improve a policy or programme as it is being implemented, while *outcome evaluation* (often called *summative evaluation*) is conducted to determine if a policy or programme works [23]. Process evaluation asks what happens along the road and explores the nature and impact of determinants for ORC, including *facilitators* as well as *barriers* [14, 17, 19]. This involves studying how the implementation was performed and received, including adaptations needed to put the intervention into action, reception among stakeholders and participants, preconditions crucial for change commitment and change efficacy and the nature and strength of resistance. Process evaluation can be conducted as pragmatic evaluation, or with more rigorous methodologies.

With regard to feasibility, evaluation should also check whether and to what degree the implementation was accomplished, such as recruitment, participation, adoption, intervention fidelity and sustainability over time. The evaluation of implementation quality may be supported by concepts such as *implementation coverage* (what proportion of the target group accomplished the intervention, and why?) and *implementation fidelity* (was the intervention, adapted by piloting, implemented according to the manuals, and how?). Confusingly, such issues are often called *implementation outcomes* [24]*.* It may be helpful to think of them as related to the implementation process itself, which is supposedly supported by already existing evidence. *Client outcomes* are also relevant endpoints for evaluation. The primary focus of a *pragmatic process evaluation* is still the implementation process itself, not necessarily including the endpoint *service outcomes* of the intervention [24].

In this sense, implementation research is very different from an intervention research study with standardized circumstances [1]. Pragmatic evaluation may nevertheless be science-informed practice, when relevant tools and standards from research with systematic and transparent reflexivity are employed. In designing the evaluation plan, we take on the role of interaction-oriented pragmatics rather than significance-oriented epidemiologists [6].

Below we present our model for small-scale implementation with pragmatic process evaluation, incorporating, summarizing and exemplifying these preconditions. A strategy for upgrading the management of depression in nursing homes in south-western Norway currently being implemented by the last author (KRI) illustrates our presentation. Applying this case before implementation and evaluation have been completed allows us to imagine and suggest more options than the finalized project will ever be able to exemplify. Hence, this is first and foremost a methodological article, not a report on an empirical study.

### The model

**Fig. 1:**
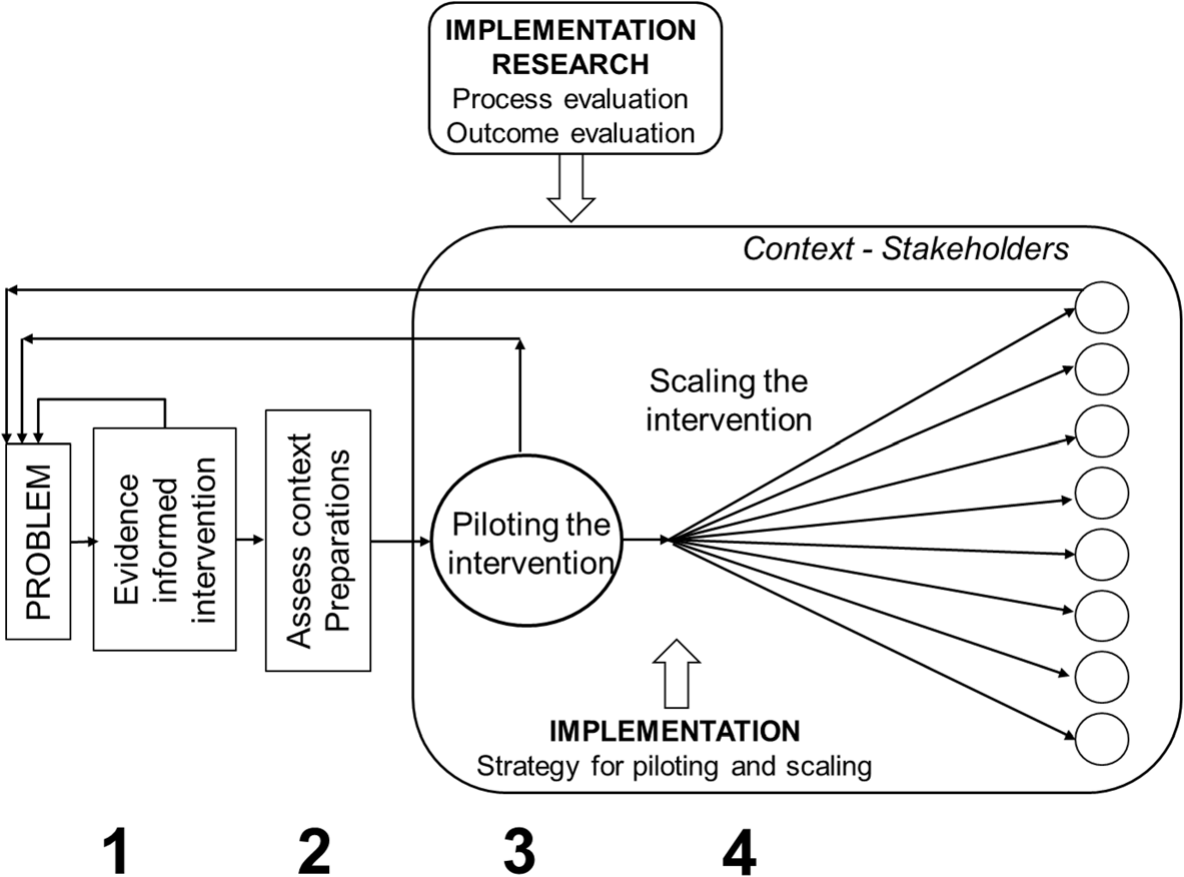
A model for implementation and implementation research

The model was designed for application in primary health care and includes implementation as well as implementation research. It consists of the following steps: 1) recognize the problem and identify a workable intervention, 2) assess the context and prepare for inception, 3) pilot the intervention on site, 4) upscale and accomplish the intervention (Fig. 1). The process is monitored by studying selected relevant aspects of experiences and outcomes from the first to the last step. In the real world, these elements are closely interwoven. To enhance understanding we present them sequentially. The model is supported by theoretical perspectives about organizational readiness for change (ORC), presented in more detail above [14].

#### 1. Recognize the problem - identify a workable intervention

A practitioner or a group of colleagues notices a problem related to procedures (or lack of such) for the diagnosis, treatment or prevention of a certain health problem in a specific practice context. A process of potential change is initiated by exploring and characterizing the *problem* at hand. Defining the nature of the problem is the starting point for developing a workable, evidence-informed intervention as a research question and potential solution. Although our model relates to a small-scale implementation, we aim for utilization beyond local problems, which may be solved by everyday practical efforts. We therefore draw attention to reflections on the extent of the problem, as well as the transferability of the chosen intervention, guided by research evidence.

In this case, the last author (KRI), a GP serving as a nursing home doctor, noticed that the use of antidepressant medication among the residents may not be optimal. Her observations were substantiated by evidence that nursing home patients use a large amount of medication in general, especially antidepressants [25, 26]. Antidepressants in the elderly are associated with serious side-effects, and the quality of treatment is crucial [27–29]. In a previous study, KRI demonstrated that doctors seldom participate in the diagnostic workup and evaluation of antidepressant treatment in the nursing home context [30, 31]. Hence, her hypothesis about haphazard management of depression in nursing homes was more than just a hunch.

When the problem has been outlined, the implementation of different *interventions* may present as a potential solution. In our model, only interventions informed by research evidence are eligible. The nature of the problem and the context of implementation determine what kind of evidence needs to be adduced. Interventions aiming for broad impact should not be implemented unless we know that they are working. Before the implementation of national guidelines for drug treatment of type 2 diabetes, we would, for example, require convincing evidence of the effect and safety of the actual drugs [32], while, for small-scale interventions, other types of evidence may be more relevant. For example, in relation to interaction strategies in primary care consultations, we might request evidence about the specific nature of the intervention and documentation about how it is accomplished. In such cases, such as implementation of home notes for patients with a long-standing illness without clinical findings, a comprehensive description of aim, context and procedures might be more useful evidence than effect studies [33].

The Cornell Scale for Depression in Dementia (CSDD) [34] is a scale for depression screening in patients with or without cognitive reduction. Five groups of depression symptoms are scored by a nurse or a relative in dialogue with the patient. The CSDD has been translated into Norwegian and is validated for use in Norwegian nursing homes [35]. KRI found CSDD to be an adequate and feasible tool for routinized depression assessment in nursing homes, as a basis for high-quality depression management and the eventual prescription of antidepressants. Based on her own experiences as a GP and a nursing home doctor, she estimated the potential for sufficient change commitment and efficacy in the actual context as reasonably good. She decided to bring her ideas into effect in real life as a small-scale implementation project, applicable in primary care and monitored by pragmatic process evaluation, to record, reflect upon and share relevant experiences.

#### 2. Assess the context - prepare for inception

Before interventions, developed in enthusiastic and experimental research settings, are turnkey and ready for utilization in a small-scale practice context, numerous steps of probing, negotiation, teaching and promotion must be performed. Greenhalgh stresses that people are far from passive recipients of innovations [36], and there is an extensive knowledge base of different theories about how human behaviour can be changed [1]. All through the process, attention must be paid to contextual circumstances. Our model encourages attention to barriers and facilitators for ORC among potential participants [14] when preparing for the implementation of an intervention in a particular health care context.

In our case, KRI first considered distributing a questionnaire to potential participants, asking about their willingness and ability to implement the intervention. However, she then decided to invite four experienced nursing home doctors [16] to a focus group-inspired discussion [37], to learn about attitudes and preconditions among potential participants in a more open-minded manner. She explored their change commitment [14], probing whether they agreed that the management of depression is an area in need of change, and whether they considered the planned intervention was beneficial and worthwhile. Furthermore, she assessed their change efficacy, by exploring their capacity to execute the planned intervention [14]. She found them sufficiently ready to “pursue the courses of action involved in change implementation”, as put by Weiner. The participants indicated that, if the implementation were to be accomplished, the management (chief nurse and medical officer) must not only support the implementation but explicitly promote it. They seemed reluctant to screen all patients with the CSDD, warning against overtreatment and time-consuming procedures. Still, they would approve routinized use of CSDD on more selected occasions. With only four participants, the data were limited but yielded substantial information, even in the absence of formal analysis.

This kind of assessment of the practice environment, first for piloting and later for scaling, is essential for planning and initial promotion. Strategic negotiations with stakeholders and collaborators, as well as the appraisal and procurement of human, social and financial resources, are also necessary. Finally, approvals regarding research ethics, confidentiality and permission to use tools and time must be secured. A *project protocol* can now be carefully elaborated. The protocol includes substantiation of the problem, a workable and evidence-informed intervention to be implemented, the aim of the implementation, suggestions about the real-life context for piloting and scaling and strategies for implementation and evaluation. Finally, resources, the time schedule, collaboration and publication plans should be briefly presented. The final version need not be laid out until the process has been under way for a while, utilizing input from experiences and interaction.

KRI assembled transcripts from the group discussion and field notes from the negotiations, constituting data for planning and an initial version of the protocol. The protocol staged the problem of antidepressant medication, motivated the evidence-based intervention with CSDD, indicated plans for piloting, scaling and evaluation and presented a time schedule. The utility of the protocol was confirmed by a successful process of permissions, funding and dialogues with the municipal health administration.

#### 3. Pilot the intervention on site

The intervention to be implemented has usually been developed in a research setting, protected from the unpredictable noise of everyday practice. We must therefore consider the evidence as a starting point, stretching and testing the transferability of the intervention by piloting it in a friendly but more normal setting. Adaptation includes sustainable simplification, with subsequent description and instruction, custom-made for the situation and the organization members involved in the intervention. The researchers need to be familiar with the research literature, including any potential critique of the intervention or its foundations.

Then it is time to negotiate with potential participants how the intervention should best be formatted to comply with their change efficacy [14], for example regarding training, practical challenges or other concerns. Establishing such a balance requires professional skills, field proficiency and research competence. However, within EBHC, the evidence is considered a binding reference, in which all core content must be preserved [1]. Furnished with the responses and adaptations on site, we elaborate the format of the intervention and summarize the vital points in a manual referring to the overall aim, context and target group and distinct specification of the intervention and how it is carried out.

By now it may also be relevant to explore expectations regarding the feasibility of later steps by studying the degree of coverage, for example by means of simple quantitative measures demonstrating the percentage of actual versus desirable or accessible participation or events. Evaluation data regarding participants’ specific experiences may be collected in different ways, for example as field notes from observation and more or less formal communication with the professionals involved, or as focus group discussions during or after the pilot period. Systematizing written documents, such as plans, proposals, budget papers or field notes from observation, may provide indications of challenges that must be considered when planning the future scaling step. The evaluator should gather potentially relevant documents, review and organize these, and – if useful – conduct a formal analysis. More often than not, such papers will offer informal but important bits and pieces of information. Knowing them, reflecting upon them and referring to them will often be more useful than qualitative analysis.

In this case, KRI established a dialogue with two nursing homes, obtaining their agreement to piloting the intervention in a total of five wards. The nursing home managements became involved and encouraged the project. Information meetings about CSDD were held, not for teaching purposes but to reinforce the involvement of the nursing home administrations [38]. CSDD would be performed by the ward nurse 1) on a routinized basis some months after patients’ admission, 2) when there were changes in functional level of the patient, or 3) when psychotropic medication was adjusted. Results would be stored in the patients’ medical record. On ward rounds, the doctor requested the results and interpreted them, together with the nurse. KRI visited the pilot sites and developed field notes about intervention experiences among the professionals involved, such as how CSDD was actually used (interval, time spent, intervention loyalty, perceived relevance, storage and utilization of results) and the impact of these issues for change commitment and change efficacy in the participating wards [14, 39]. She observed that an adequately staffed ward with a dedicated doctor who collaborates closely with the nurses and finds CSDD helpful constitutes the most advantageous environment for implementation. KRI was also exploring the preconditions for and likelihood of the sustainability of the intervention beyond the pilot period. Alongside practicalities, she reflected on which aspects of the process to prioritize regarding evaluation and data collection during the scaling step.

#### 4. Upscale and organize the intervention

After piloting, the modified intervention is ready for *upscaling*, which means implementation to a broader context. This step requires social and organizational skills, because the intervention will not be taken on board by participants and end users until they are sufficiently informed, motivated, trained and ready for change [6, 14]. Grol et al. emphasize that systematic approaches and thorough planning, assessment of the intervention as a useful product, preparedness in the target group and a diagnostic analysis of target group, context and feasibility are required for successful implementation, preferably within already existing structures [17]. They also draw attention to the involvement of the target group in planning, development and adjustment, as well as a continuous evaluation of process and outcome [17]. Based on a thorough literature review, Greenhalgh recommends systematic assessment of whether a specific innovation is right for the organization, judicious attention to project management, valuing and supporting organizational sense-making, and studying organizational routines and the interaction between them, as well as the systematic encouragement of links beyond the organization [1].

Strategies for recruitment regarding locations and number of sites are also important considerations for upscaling. We should include not only the very enthusiastic, but also those who are somewhat reluctant. Moreover, careful assessment of available resources is especially important in a small-scale implementation project. The project can always be expanded later, if the initial efforts appear to be successful. Implementation is of no use if the intervention is forgotten a year later. Do not forget to invest in strategies that enhance sustainability.

Priorities must also be set regarding strategies for process evaluation during the upscaling step. Simple measures for coverage proportion (see above) can offer important clues for assessment of the implementation process. Focus group interviews or participant observation can complement numerical data, providing further insights into facilitators or barriers with an impact on achievement. The latter approaches can also be used to learn more about organizational processes, interactions and experiences. Intervention loyalty may be studied by qualitative or quantitative methods, depending on the intended level of standardization of the intervention. In a small-scale implementation project, the number of aspects to be included in the evaluation plan should not be excessive. It is advisable to try to explore a few selected indicators leading to information of special relevance for colleagues, to whom you hope to spread the good idea. Then you conduct an adequate collection and analysis of relevant data for this purpose.

In this case, KRI planned upscaling of the intervention, starting with strategies for recruitment. After obtaining permits and support from relevant authorities, she arranged meetings for nursing home doctors in neighbouring municipalities with lectures about the management of depression, presenting CSDD as a workable tool to support diagnostic routines in the nursing homes. She enlisted five wards from three nursing homes. One of the wards from the pilot sites also volunteered to join the next step of implementation. At this point of the process, KRI observed that implementation of CSDD was achievable. Several participants found CSDD helpful, verifying or adjusting their clinical assessment, especially with previous scores available for comparison. Some of them also mentioned positive side-effects of the new routines, such as increased attention to various depressive symptoms or to the impact of environmental support. KRI also wanted to take advantage of experiences from the scaling step to learn more about preconditions for coverage, maintenance and sustainability beyond individual commitment. She planned another round of participant observation, in which particular preconditions for intervention coverage and loyalty could be explored on site [39]. She also considered a mixed-methods project about the clinical consequences of the intervention [40], based on selected data from patients’ medical records and interviews with participating staff.

It turned out, however, that such a plan would require more resources than were available within the time available. Instead, she planned to complement the fieldwork with a purposive sample of three focus groups, highlighting contextual facilitators and barriers regarding organizational readiness for change during the process and in the future. In this way, the future sustainability of the intervention could be assessed and encouraged.

Finally, KRI considered the possibilities of including relevant outcome measures in the evaluation plan, such as clinical effectiveness (better depression management) after the intervention. She concluded, however, that such an ambition would require a pre-planned randomized controlled trial design with many more participating sites than she would be able to handle at this point. KRI reported this idea to the research unit with which she is connected, hoping for a future Ph.D. student to team up with her. An evaluation of service users’ satisfaction related to the intervention could also be included in her plan. KRI concluded that her evaluation plan still captured the most important issues regarding process and context for further implementation efforts.

## Discussion

Advocating EBHC in the primary care context, we developed a model for small-scale implementation accompanied by pragmatic process evaluation, in which relevance and feasibility were prioritized elements. This methodological article presents the principles and practical application of conduct and evaluation, setting out the four steps of the model, illustrated by a real-life example. Below, we discuss the impact of this model, including its strengths and weaknesses.

### What is known from before? – what does this model add?

The model presented here was developed by GPs with clinical experiences from comparably small primary care organizations. Compared with the dimensions and structure of a hospital setting, primary care is more homogeneous and transparent, with fewer levels than specialist care organizations. Furthermore, we were not strangers from professional implementation companies but colleagues who were familiar with the context and working conditions of the place in which the intervention was to be implemented. Peer-driven implementation and evaluation are probably more common in primary care, because practitioners themselves realize specific needs for quality improvement and are often in charge of devising bottom-up strategies for change. Limited funding may inspire creative strategies and designs suited for everyday practice, emphasizing relevance and flexibility corresponding to Peters’ recommendations [6]. Under such conditions, organizational readiness for change may be better accessible than in a large and complex organization, where assessment and the influence of change commitment and change efficacy require more professional investment. Nevertheless, our model can also be considered generic and thus applicable in small-scale settings beyond primary care.

We are not the first to suggest that patient care can be improved by the implementation of evidence-based interventions [6, 17, 19, 41]. There is already a vast amount of literature available about theories and strategies for behavioural change among individual professionals and their organizational contexts [1]. Furthermore, implementation research has in the last decades become a sub-discipline within health care systems research, offering and expecting ambitious and advanced methodological tools for the planning, monitoring and evaluation of implementation projects [16, 19, 42]. Among our colleagues, we have noticed that these trends seem to discourage innovative practitioners from initiating noteworthy ideas for implementation.

Greenhalgh and Wieringa suggest that research should leave behind the focus on a ‘know–do gap’ in order to create space for practical wisdom, tacit knowing, the complexities of the relationship between knowledge and power and enhance collaboration between research, policy decisions and clinical practice [43]. Implementation research is embedded in reality, and practitioners (rather than researchers) may articulate the problem and ask relevant questions as a starting point for new thinking [6].

In small-scale projects, implementation as well as implementation research are conducted by the same person, often an innovative enthusiast with a strong belief in the intervention. Methodological skills are therefore essential, especially the ability to establish an analytic distance from the field of action [44]. A pragmatic evaluation is not a casual and haphazard process but a science-informed strategy, in which transparency and systematic, critical reflection are supported by methodological skills. An important difference from a traditional research project is the meticulous priorities identified to assign resources for description and discussion of the processes according to contextual relevance.

Endorsing the idea that implementation research is science-informed practice [1], our model incorporates several distinctive features intended to encourage small-scale implementation projects in real life. First, the model – referring to EBHC – implies that research evidence about the intervention already exists: we do not start from scratch. In our example, KRI did not create the intervention, because it had already been designed, validated and published. Second, we do not confine our concept of evidence to effect. The relevance aspect of different kinds of available evidence, given the ambitions and the context of the project, is more important than choosing a specific design. In our example, CSDD was chosen because this tool was appropriate and simple. Third, we prioritize elaboration and adaptation of the intervention to match the context, rather than using a standardized version from a research setting. In our example, the manual for practical application of CSDD was elaborated in the pilot step. Fourth, we aim for evaluation methods with a capacity for systematic and transparent reflection, prioritizing selected issues relevant for sharing experiences from the process. In our example, a few low-key approaches for the collection and analysis of relevant data were matched by the limited resources available to the project, although they still provided useful information about ORC [14] to be used and shared.

### Does the intervention work?

Cost-benefit logic implies that resources should not be wasted on interventions that do not work. The default image of a randomized controlled trial (RCT) readily comes to the mind of health care professionals when discussing intervention projects. There are, however, important differences between projects testing the effect of an intervention within the standardized context of an RCT and projects putting evidence from previously documented research into action within a complex real-life situation. In health care services research, the question of whether something works or not cannot be answered with a simple yes or no. The framing of the answer depends on the aim, context, outcome measures and other vital preconditions.

Referring to Cochrane [45], Rothwell asks the crucial question about the external validity of RCTs: “To whom do the results of this trial apply?” [46]. Consequently, conceptual accuracy is needed to evaluate the outcome of an intervention, distinguishing between *efficacy* (whether an intervention produces the expected result under ideal circumstances) and *effectiveness* (the degree of beneficial effect in “real-world” clinical settings) [47].

Still, our model does not include a mandatory element of effectiveness evaluation. Process evaluation is emphasized and given priority in the model, aiming for clear descriptions of the adapted intervention and experiences from putting it into action. This is because process evaluation is a logical precondition for outcome evaluation: it makes no sense to attempt to assess the efficacy or the effectiveness of a “black box” intervention [48] whose content is not distinctly indicated. For the evaluation of a small-scale intervention, knowledge about the nature and content of the intervention under real-life conditions, and how it was taken from knowledge to action, must come first. Was the intended intervention actually implemented, and in what format?

Our model conveys certain preferences that were specifically elaborated for a small-scale primary care context – a distinctive trait of the context our model is intended to serve. This prioritization does not imply that we consider outcome evaluation to be of no great concern. It is of course interesting and useful to find out to what degree outcome measures from the research context can be obtained in a real-life context. A small-scale implementation project might therefore grow to medium or large format, in which such evaluation is included, for example in the design of a pragmatic RCT [49].

## Conclusion

Implementation research deserves a model consistent with the specific characteristics of the actual health care context. The inevitable uncertainty and unpredictability of primary health care have here been incorporated into a flexible and manageable approach, in which context and limited resources are considered. In finding an appropriate middle ground between rigour and flexibility, some compromises must be made and some ambitions must be balanced. Presenting this model specifically designed for small-scale implementation research, we remain grounded as practitioners. The model recognizes the skills of practical knowing as something other than traditional medical research, while also maintaining academic values such as systematic and transparent reflection, with adequate tools. Considering the purpose and the context of our model, we argue that these methodological priorities, emphasizing relevance and feasibility, are strengths, and not limitations.

## Abbreviations

CSDD: The Cornell Scale for Depression in Dementia
EBHC: Evidence-based health care
EBM: Evidence-based medicine
GP: General practitioner
KRI: Kristina Riis Iden (the last author)
ORC: Organizational Readiness for Change
Ph.D.: Philosophiae Doctor
RCT: Randomized controlled trial

## References

[1] Greenhalgh T (2018). How to implement evidence-based healthcare.

[2] Ioannidis JP (2016). Why most clinical research is not useful. PLoS Med.

[3] Pathirana T, Clark J, Moynihan R (2017). Mapping the drivers of overdiagnosis to potential solutions. BMJ.

[4] Vaccarella S, Franceschi S, Bray F, Wild CP, Plummer M, Dal Maso L (2016). Worldwide thyroid-cancer epidemic? The increasing impact of overdiagnosis. N Engl J Med.

[5] Grol R, Baker R, Moss F. Quality improvement research: the science of change in health care. In: Grol R, Baker R, Moss F, editors. Quality improvement research: understanding the science of change in health care. London, BMJ Books; 2004. p. 1–5. 10.1136/qhc.11.2.110 PMC1743590 12448794

[6] Peters DH, Tran NT, Adam T: Implementation research in health: A practical guide. In. Geneva: Alliance for Health Policy and Systems Research, World Health Organization; 2013. http://who.int/alliance-hpsr/alliancehpsr_irpguide.pdf ( Accessed 23.Sept.2015).

[7] Malterud K (1995). The legitimacy of clinical knowledge: towards a medical epistemology embracing the art of medicine. Theor Med.

[8] Malterud K (2001). The art and science of clinical knowledge: evidence beyond measures and numbers. Lancet.

[9] De Maeseneer JM, van Driel ML, Green LA, van Weel C (2003). The need for research in primary care. Lancet.

[10] Carlsen B, Glenton C, Pope C (2007). Thou shalt versus thou shalt not: a meta-synthesis of GPs’ attitudes to clinical practice guidelines. Br J Gen Pract.

[11] Lugtenberg M, Zegers-van Schaick JM, Westert GP, Burgers JS (2009). Why don't physicians adhere to guideline recommendations in practice? An analysis of barriers among Dutch general practitioners. Implement Sci.

[12] Austad B, Hetlevik I, Mjolstad BP, Helvik AS (2016). Applying clinical guidelines in general practice: a qualitative study of potential complications. BMC Fam Pract.

[13] Flottorp S, Oxman AD, Håvelsrud K, Treweek S, Herrin J (2002). Cluster randomised controlled trial of tailored interventions to improve the management of urinary tract infections in women and sore throat. BMJ.

[14] Weiner BJ (2009). A theory of organizational readiness for change. Implement Sci.

[15] Craig P, Dieppe P, Macintyre S, Michie S, Nazareth I, Petticrew M (2008). Developing and evaluating complex interventions: the new Medical Research Council guidance. BMJ.

[16] Peters DH, Adam T, Alonge O, Agyepong IA, Tran N (2013). Implementation research: what it is and how to do it. BMJ.

[17] Grol R, Wensing M, Eccles M (2005). Improving patient care. The implementation of change in clinical practice.

[18] Graham R, Mancher M, Wolman DM, Greenfield S, Steinberg E (2011). Clinical practice guidelines we can trust Washington: Institute of medicine, The National Academies Press.

[19] Fixsen DL, Naaom SF, Blase KA, Friedman RM, Wallace F (2005). Implementation research: a synthesis of the literature..

[20] Patton MQ (2008). Utilization-focused evaluation.

[21] Haraway D, Haraway D (1991). Situated knowledges; the science question in feminism and the privilege of partial perspective. Simians, cyborgs, and women The reinvention of nature.

[22] Östlund U, Kidd L, Wengström Y, Rowa-Dewar N (2011). Combining qualitative and quantitative research within mixed method research designs: a methodological review. Int J Nurs Stud.

[23] Patton MQ (2015). Qualitative research & evaluation methods: integrating theory and practice.

[24] Proctor E, Silmere H, Raghavan R, Hovmand P, Aarons G, Bunger A, Griffey R, Hensley M (2011). Outcomes for implementation research: conceptual distinctions, measurement challenges, and research agenda. Admin Pol Ment Health.

[25] Nygaard HA, Ruths S, Straand J, Naik M (2004). Not less but different: psychotropic drug utilization trends in Norwegian nursing homes during a 12-year period. The Bergen District nursing home (BEDNURS) study. Aging Clin Exp Res.

[26] Onder G, Liperoti R, Fialova D, Topinkova E, Tosato M, Danese P, Gallo PF, Carpenter I, Finne-Soveri H, Gindin J (2012). Polypharmacy in nursing home in Europe: results from the SHELTER study. J Gerontol A Biol Sci Med Sci.

[27] Coupland C, Dhiman P, Morriss R, Arthur A, Barton G, Hippisley-Cox J (2011). Antidepressant use and risk of adverse outcomes in older people: population based cohort study. BMJ.

[28] Bakken MS, Engeland A, Engesaeter LB, Ranhoff AH, Hunskaar S, Ruths S (2013). Increased risk of hip fracture among older people using antidepressant drugs: data from the Norwegian prescription database and the Norwegian hip fracture registry. Age Ageing.

[29] Banerjee S, Hellier J, Dewey M, Romeo R, Ballard C, Baldwin R, Bentham P, Fox C, Holmes C, Katona C (2011). Sertraline or mirtazapine for depression in dementia (HTA-SADD): a randomised, multicentre, double-blind, placebo-controlled trial. Lancet.

[30] Iden KR, Hjorleifsson S, Ruths S (2011). Treatment decisions on antidepressants in nursing homes: a qualitative study. Scand J Prim Health Care.

[31] Iden KR, Engedal K, Hjorleifsson S, Ruths S (2014). Prevalence of depression among recently admitted long-term care patients in Norwegian nursing homes: associations with diagnostic workup and use of antidepressants. Dement Geriatr Cogn Disord.

[32] Helsedirektoratet. Nasjonale faglige retningslinjer: Diabetes - Forebygging, diagnostikk og behandling. In: [National guidelines: Diabetes - prevention, diagnosis and treatment] 15/1674 (in Norwegian). https://helsedirektoratet.no/retningslinjer/diabetes. (Accessed 28 Aug 2016).

[33] Stensland P, Malterud K (1997). New gateways to dialogue in general practice. Development of an illness diary to expand communication. Scand J Prim Health Care.

[34] Alexopoulos GS, Abrams RC, Young RC, Shamoian CA (1988). Cornell scale for depression in dementia. Biol Psychiatry.

[35] Barca ML, Engedal K, Selbaek G (2010). A reliability and validity study of the Cornell scale among elderly inpatients, using various clinical criteria. Dement Geriatr Cogn Disord.

[36] Greenhalgh T, Robert G, Macfarlane F, Bate P, Kyriakidou O (2004). Diffusion of innovations in service organizations: systematic review and recommendations. Milbank Q.

[37] Morgan D (1997). Focus groups as qualitative research.

[38] Rosemond CA, Hanson LC, Ennett ST, Schenck AP, Weiner BJ (2012). Implementing person-centered care in nursing homes. Health Care Manag Rev.

[39] Van Manen M (1990). Researching lived experience : human science for an action sensitive pedagogy.

[40] Creswell JW, Clark VLP (2011). Designing and conducting mixed methods research.

[41] Woolf SH (2008). The meaning of translational research and why it matters. JAMA.

[42] Eccles MP, Mittman BS (2006). Welcome to implementation science. Implementation Science.

[43] Greenhalgh T, Wieringa S (2011). Is it time to drop the ‘knowledge translation’ metaphor? A critical literature review. J R Soc Med.

[44] Finlay L, Finlay L, Gough B (2008). Introducing Reflexivity. Reflexivity - A practical guide for researchers in health and social sciences.

[45] Cochrane AL (1972). Effectiveness and efficiency: random reflections on health services.

[46] Rothwell PM (2005). External validity of randomised controlled trials: “to whom do the results of this trial apply?”. Lancet.

[47] Gartlehner G, Hansen RA, Nissman D, Lohr KN, Carey TS (2006). Criteria for distinguishing effectiveness from efficacy trials in systematic reviews. Technical Reviews.

[48] Glasziou P, Meats E, Heneghan C, Shepperd S (2008). What is missing from descriptions of treatment in trials and reviews?. Br Med J.

[49] Harvey G, Wensing M (2003). Methods for evaluation of small scale quality improvement projects. Quality & safety in health care.

